# 

**Published:** 1868-06-15

**Authors:** 


### CHICAGO MEDICAL JOURNAL.

**Edited by J. ADAMS ALLEN, M.D., LL.D.,**

Professor of Principles and Practice of Medicine and Clinical Medicine in Rush Medical College.

All letters for the JOURNAL should be addressed to Dr. J. ADAMS ALLEN P. O. Box 1948, Chicago, Ill.

TERMS — $3 Per Annum, in Advance;

$4 if not paid before 1st of July, in each year. Single copies, 25 cents.

